# *Shewanella* infection in humans: Epidemiology, clinical features and pathogenicity

**DOI:** 10.1080/21505594.2022.2117831

**Published:** 2022-09-05

**Authors:** Keyi Yu, Zhenzhou Huang, Yue Xiao, Duochun Wang

**Affiliations:** a State Key Laboratory of Infectious Disease Prevention and Control, National Institute for Communicable Disease Control and Prevention, Chinese Center for Disease Control and Prevention (China CDC), Beijing, China; b Center for Human Pathogenic Culture Collection, China CDC, Beijing, China

**Keywords:** *Shewanella* spp., *Shewanella* infection, epidemiology, clinical features, pathogenicity, virulence

## Abstract

The genus *Shewanella* consists of Gram-negative proteobacteria that are ubiquitously distributed in environment. As the members of this genus have rapidly increased within the past decade, several species have become emerging pathogens worldwide, attracting the attention of the medical community. These species are also associated with severe community- and hospital-acquired infections. Patients infected with *Shewanella* spp. had experiences of occupational or recreational exposure; meanwhile, the process of infection is complex and the pathogenicity is influenced by a variety of factors. Here, an exhaustive internet-based literature search was carried out in PUBMED using terms “*Achromobacter putrefaciens*,” “*Pseudomonas putrefaciens*,” “*Alteromonas putrefaciens*” and “*Shewanella*” to search literatures published between 1978 and June 2022. We provided a comprehensive review on the epidemiology, clinical features and pathogenicity of *Shewanella*, which will contribute a better understanding of its clinical aetiology, and facilitate the timely diagnosis and effective treatment of *Shewanella* infection for clinicians and public health professionals.

## Introduction

The genus *Shewanella* comprises Gram-negative, facultative anaerobic, oxidase-positive and motile bacteria [1,2]. Due to the unique physiological and respiratory versatility, *Shewanella* spp. can survive in a wide range of ecological niches (for example, suboptimal environmental conditions with extreme salinity and high barometric pressure, spoilt foods and clinical specimens) and have been applied in environmental protection and industrial development [2]. Since the first identification of *Shewanella putrefaciens* in 1931 [3], several *Shewanella* species have recently emerged as worldwide pathogens, attracting the attention of the medical community [4]. Species like *Shewanella algae*, *Shewanella putrefaciens*, and *Shewanella xiamenensis* [5,6] have been proven to associate with human [4,7] and aquatic livestock diseases [8–11]. Also, *Shewanella* spp. can be found in food processing and storage [7]. Nowadays, members of the genus *Shewanella* are more than 70 (http://www.bacterio.net/shewanella.html). In order to identify the clinical features and to evaluate resistance pattern of *Shewanella* species, Wincy et al. retrospectively analyzed demographics, antibiotics, microbiology, and outcomes of the 128 patients who has been admitted to a regional hospital in Hong Kong with *Shewanella* species infection from 1^st^ April 2010 to 31^th^ December 2020 [4]. In this review, we searched for case reports of *Shewanella* spp. infections in PUBMED from 1978 to June 2022 and provided an overview of the epidemiological features, clinical manifestations and pathogenicity of *Shewanella* infection, which may be helpful in guiding treatment strategy determinations and providing responsive therapy.

## Epidemiology

In sharp contrast to the beneficial effects of *Shewanella*, occupational or recreational exposure is the two most common routes of infection [7]. The physiological versatility of the genus *Shewanella* allows for its wide distribution. As mentioned above, *S. putrefaciens* was originally isolated from rotten butter, and since then, it has been identified as a food spoiler in several foods, including poultry, beef and seafood [7]. Other *Shewanella* species are mainly found in marine environments [12], and *S. algae*, *S. putrefaciens*, and *S. xiamenensis* have become emerging opportunistic pathogens. The *Shewanella*-related infections are sporadically reported and many cases are being documented. All published literatures about the case reports of *Shewanella* infection have been systematically reviewed to summarize the demographic information and clinical characteristics [3,7,13–18]. A total of 125 studies involving 273 patients were included for final analysis.

### Geographical distribution

**Figure 1.**
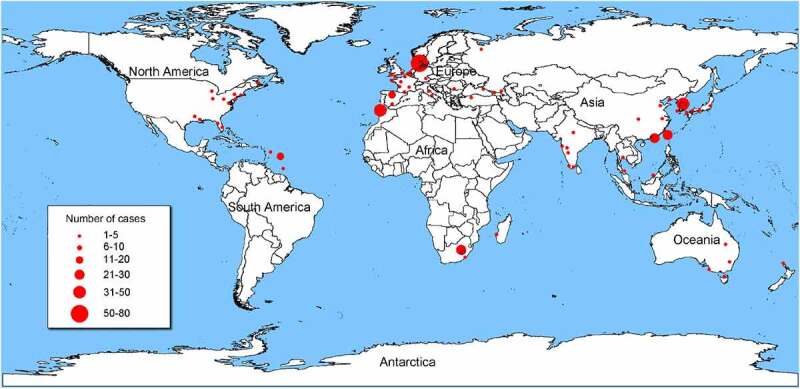
Geographical distribution of 273 *Shewanella* infectious cases. The geographical distribution of cases are Denmark (n = 71), Spain (n = 39), Africa (n = 30), China (n = 19), U.S.A (n = 17), Martinique (n = 16), India (n = 12), France (n = 9), Japan (n = 7), Korea (n = 5), Turkey (n = 6), Australia (n = 5), Caucasian (n = 3), Croatia (n = 2), Malaysia (n = 2), Thailand (n = 2), Belgium (n = 2), Italy (n = 2), Panama (n = 1), Mexico (n = 1), Moroccan (n = 1), Belize (n = 1), Wakefield (n = 1), Virginia (n = 1), Bahamas (n = 1), Romania (n = 1), Madagascar (n = 1), Germany (n = 1), Caribbean (n = 1), Cyprus (n = 1), UK (n = 1), Brunei Darussalam (n = 1), Puerto Rico (n = 1), Russia (n = 1), New Zealand (n = 1), Côte d’Ivoire (n = 1), and Israel (n = 1). Information on the geographical location of 5 cases is not available.

Cases of *Shewanella* infection have been reported around the world. Places with hot summer weather have the highest number of reported cases (Figure 1), e.g. Southern Europe (n = 71), Southeast Asia (n = 48), and Southern Africa (n = 30). Most reported cases were from tropical, subtropical, and temperate countries such as Australia, Belgium, Denmark, Israel, Spain, and Turkey [12]. Coastal cities and regions suitable for tourism and living, like Taiwan of China, Martinique, Barbados, Canary Islands, also have many cases. The prevalence of *Shewanella* infection varies greatly in different geographical locations, being correlated with the temperature of seawater and the frequency of strain occurrence [19].

### Population distribution and species composition

The ages of people infected with *Shewanella* ranged from neonates to 92 years old (Table 1). Among all the cases, the elderly (over 60 years old) accounted for the largest proportion (34.43%) compared to other age groups. Apart from patients with undisclosed sex (39.56%), the ratio of male to female was 2.84:1(122/43). In the study of Wincy et al., 61.7% of the 128 patients were male, with an average age of 78 [4]. In terms of age and gender composition, we have reached the same conclusion. The reason for this may be that men are more likely to engage in occupations or activities related to marine habitats, including fishing and diving. Clinical strains of *Shewanella* can be isolated from samples such as blood, sputum, urine, and intra-abdominal. The species compositions of clinical *Shewanella* infection were *S. algae* (35.16%), *S. putrefaciens* (28.94%), and *S. xiamenensis* (0.37%). Some studies failed to provided definitive information for accurate species identification. In most of the cases (35.16%), *Shewanella* was a member of the multi-microbial infections, making it difficult to explain its exact role in pathogenicity and disease progression.

**Table 1.** Clinical characteristics associated with *Shewanella* infection.

Characteristic	Detailed information	n (%)
Gender	Male	122 (44.69)
	Female	43 (15.75)
	Gender ambiguity	108 (39.56)
Age composition	Juvenile (newborn-14)	60 (21.98)
(Neonates–92 years)	Meridian of life (15–60)	82 (30.04)
	The elderly (>60)	94 (34.43)
	Age ambiguity	37 (13.55)
Infectious pathways	Underlying Disease	193 (70.70)
	Environmental exposure	119 (43.59)
	Trauma	28 (10.26)
	Substandard living and Poor nutrition	17 (6.23)
Primary presentation or diagnosis	Bloodstream infections	101 (37.00)
	Skin and soft tissue infections	98 (35.90)
	E.N.T Disorders	75 (27.47)
	Intra-abdominal	25 (9.16)
	Bone Arthropathy	18 (6.60)
	Chest infection	12 (4.40)
	CNS disease	6 (2.20)
	Cardiovascular	5 (1.83)
Isolate	*S. algae*	96 (35.16)
	*S. putrefaciens*	79 (28.94)
	*S. xiamenensis*	1 (0.37)
	*Shewanella* sp.	1 (0.37)
	polymicrobial	96 (35.16)

According to the available case reports, *S. algae* and *S. putrefaciens* were most frequently isolated from blood samples and skin injury swabs, which is consistent with the conclusion drown by Janda [7]. It is worth noting that in Denmark, *S. algae* has also been isolated in the pure culture of the ear swabs of 33 patients, suggesting its important role in causing ear infections [15]. Moreover, *S. putrefaciens* has been isolated from patients suffered from peritonitis [20–24] and meningitis [25], and *S. xiamenensis* has been isolated from intestinal specimen [6].

### Infectious pathways and influencing factors

The infection caused by the genus of *Shewanella* is complex and can be influenced by a variety of factors. Firstly, *Shewanella* spp. are abundantly distributed in water environments, providing ample opportunities for these bacteria to come into close contact with humans [7]. Recreation (e.g. diving, playing on the beach), occupational exposure (e.g. crabbing, fishing), seafood ingestion, puncture wounds caused by marine organisms (sea urchins, fish), or the direct exposure of a wound to aquatic environments can increase the risk of *Shewanella* infection. The percentage of exposure to marine environments was reported to be 43.59% among patients with *Shewanella* infection (Table 1). Secondly, *Shewanella* infections are commonly found in patients with immunocompromised state, including malignancies, severe heart failure, renal failure, hepato-biliary disease, neutropenia, and chronic ulcerations on the lower extremities [7,26], although infections in healthy individuals with no medical history had also been reported [13,27–30]. This kind of microorganisms can also be cultured from the clinical samples of burned or multiply traumatized patients, and patients with diabetes, leukaemia or immunosuppressive therapy. Among the above mentioned, liver diseases appeared to be a strong risk factor [23,31–34]. Thirdly, *Shewanella* spp. have been found to associate with cases of nosocomial infection, leading to the outbreaks of healthcare-associated infections [5,24,35–37]. Invasive procedures like catheterization and intubation are also an important source of infection [22,24,38]. An outbreak of 31 cases of abdominal and biliary tract infections or bacteraemia of *Shewanella*, caused by the exposure to a shared measuring cup, was reported in a general surgery unit in South Korea [35]. In addition, the differences of development, living styles and environmental pollution conditions result in different incidence rates of *Shewanella* infection. Brink et al. reported 28 cases of bacteraemia caused by *S. putrefaciens* in South Africa, and almost all cases were related to poor hygiene [16].

## Clinical features and treatment

### Symptom classification

**Figure 2.**
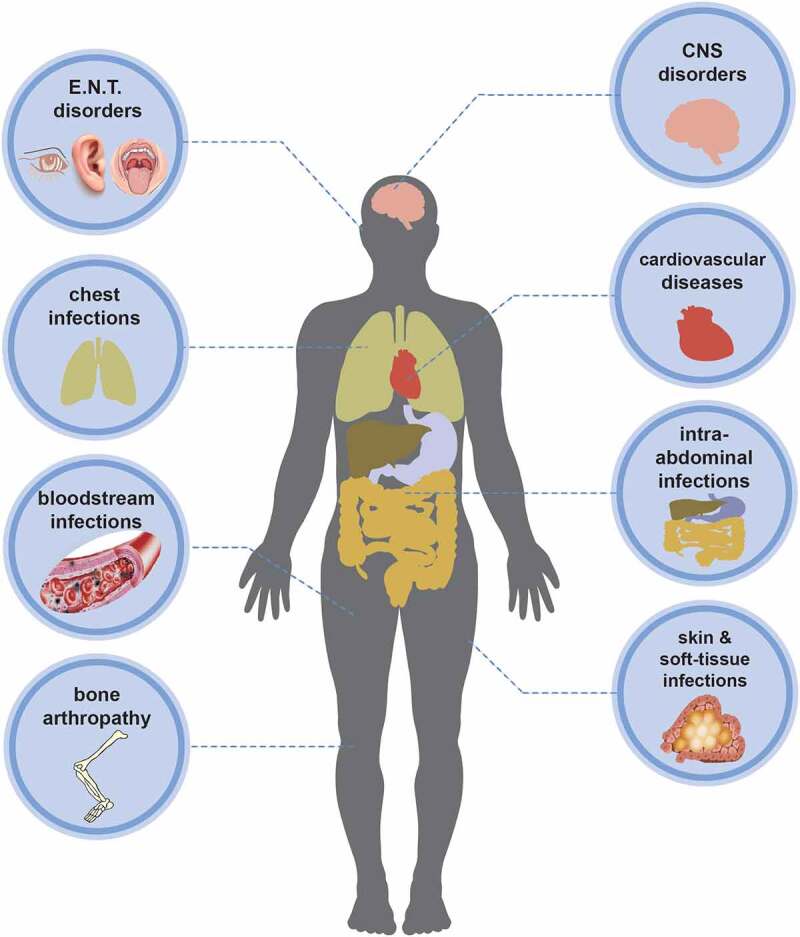
Clinical infections and diseases caused by *Shewanella* species.

As opportunistic pathogens, *Shewanella* spp. can cause a wide spectrum of clinical diseases in human. Documented illnesses by Janda et al. showed that *Shewanella*-related syndromes can be divided into five categories, including skin and soft-tissue infections (SSTIs), invasive diseases (such as sepsis), hepatobiliary diseases (hepatocirrhosis, liver cancer, cholangitis), otitis media and associated sequelae, and other infection [3]. SSTIs, including cellulitis, abscess, or necrotizing fasciitis, were considered as the most common clinical manifestation of infection. We divided *Shewanella*-related diseases into eight categories according to the infection site (Figure 2), including ear, nose, and throat (E.N.T) disorders, central nervous system (CNS) disorders, chest infections, cardiovascular diseases, bloodstream infections (bacteraemia, septicaemia), intra-abdominal infections, bone arthropathy, skin and soft-tissue infections (SSTIs). The first four common clinical manifestations consist of bloodstream infections, SSTIs, E.N.T disorders, and intra-abdominal infections (Table 1). Certain bone or joint diseases such as arthritis [13,38,39], osteomyelitis [40–43], and discitis [44] can also be caused by *Shewanella*. SSTIs and bloodstream infections were reported to be predominate in the 16 cases of *Shewanella* infection in hospitals in Taiwan, followed by biliary tract infections [14]. Consistent with the conclusion of Vignier et al., bloodstream infection was the most common complication caused by *Shewanella* infection [13]. Bacteraemia was not associated with the ear infection of *Shewanella* in any case published to date.

### Underlying diseases

Among the patients with *Shewanella* infection, 70.70% of them (n = 193) had underlying diseases (Table 2), some even had multiple diseases. As Wincy et al. concluded, hepatobiliary diseases, malignancy, chronic kidney disease or end-stage renal failure, and diabetes mellitus are important underlying disease [4] (Table 2). In our study, 31 patients (11.36%) had hepatobiliary, spleen and pancreatic related diseases, like hepatitis, cirrhosis, liver abscess, and gallstones. It needs to be acknowledged that, with the increase in the number of included cases, the status of E.N.T disorders in the underlying diseases cannot be ignored [15]. Cancer infiltration, tumour block compression and other mechanical compression bring about neurological lesions and then result in joint diseases. *Shewanella* spp. are often found in bile as a part of mixed flora [35,45]. Biliary tract infection and cholelithiasis are mutually causal. Biliary tract obstruction caused by cholelithiasis will lead to cholestasis and bacterial reproduction. Diabetic microvascular lesions can affect the healing of foot ulcers, leaving the wound exposed and prone to bacterial infection [46]. Among the patients with *Shewanella* infection, 30 (10.99%) were diabetics with the complication of lower limb soft tissue ulcer(Table 2). In addition to malignant tumours, kidney disease, diabetes and hepatobiliary diseases, respiratory tract-related diseases like pneumonia, tuberculosis, and chronic obstructive pulmonary disease are also considered to associate with *Shewanella* infection. Erfanmanesh et al. reported a mixed infection of *Streptococcus dolphins* and *S. algae* in 2019. They concluded that the systemic *Streptococcosis* may trigger the formation of ulceration by *Shewanella* and highlighted the potential significance of *Shewanella* as a pathogen to cause pulmonary oedema and concomitant infection [47].

**Table 2.** Summary report of the underlying diseases of *Shewanella* infection.

Category of underlying diseases	Detailed diseases	Case number (%)	Infectious Diseases	Bacteria
Chronic wasting disease	E.N.T disorders	55 (20.15)	Sepsis, intraabdominal infection, skin and soft tissue infections, bone arthropathy, E.N.T disorders, chest infections, cardiovascular disease	*S. putrefaciens, S.haliotis, S. algae, S. xiamenensis, Enterococcus faecalis, Escherichia coli, Aeromonas sobria, Klebsiella pneumoniae, Bacteroides fragilis, Staphylococcus aureus, Enterococcus casseliflavus, Bacteroides uniformis, Citrobacter freundii, Aeromonas sobri*
	Hepatobiliary, spleen and pancreatic related diseases	31 (11.36)		
	Heart disease	32 (11.72)		
	Diabates	30 (10.99)		
	Hypertension	28 (10.26)		
	Kidney disease	23 (8.42)		
	Tumour	17 (6.23)		
	Dyslipidemia	15 (5.49)		
	Bone Arthropathy	14 (5.13)		
	Vascular disease	10 (3.66)		
	Pulmonary disease	9 (3.30)		
Infectious disease	Lepriasis	3 (1.10)	Skin and soft tissue infections	*S. putrefaciens*
	Infected with virus	1 (0.37)		
	Infected with bacteria	1 (0.37)		
Immune deficiency	Primary immunodeficiency	16 (5.86)	Skin and soft tissue infections, Sepsis	*S. putrefaciens, Mycobacterium marinum, S. algae*
	Systemic lupus erythematosus (SLE)	1 (0.37)		
Neonatal diseases	Respiratory distress	17 (6.23)	Sepsis	*S.putrefaciens, Pseudomonas aeruginosa, Enterococcus faecalis, Morganella morganii, Serratia marcescens, Escherichia coli, Enterobacter cloacae, Flavobacterium odoratum, Klebsiella pneumoniae*
	Infectious pneumonia	4 (1.47)		
	Congenital syphilis	2 (0.73)		
	Patent ductus arteriosus	1 (0.37)		
	Congenital hydrocephalus	1 (0.37)		
	Premature rupture of foetal membranes	1 (0.37)		
Nervous system disease	Parkinson	1 (0.37)	Sepsis	*S. algae, S. putrefaciens*
	Epilepsy	1 (0.37)		

### Identification

**Figure 3.**
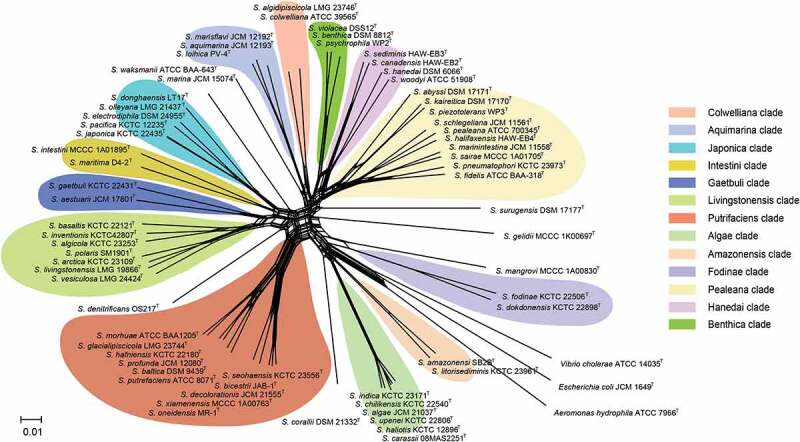
Concatenated split network tree based on six genes loci. The *gyrA*, *gyrB*, *infB*, *recN*, *rpoA*, and *topA* gene sequences from 66 *Shewanella* species were concatenated and reconstructed using SplitsTree program (version: 4.14.4), based on Jukes-Cantor model.

Most species in the genus of *Shewanella* are non-fermentable, with the same phenotypic characteristics; as a result, there are limited biochemical indexes to distinguish them at the species level. Meanwhile, existing clinical commercial analysis systems, such as API 20E, API 20 NE, Vitek 2 GN card (bioMérieux, France) and matrix-assisted laser desorption/ionization time-of-flight mass spectrometry (MALDI-TOF MS), cannot correctly identify *Shewanella* at the species level [7,48] due to the limited information of species (like *S. algae* and *S. putrefaciens*) in databases. Our previous study, which based on the newly constructed peptide mass reference spectra database, containing 36 *Shewanella* species, demonstrated that *Shewanella* isolates can be effectively distinguished according to their different MS fingerprintings [49], indicating that MALDI-TOF MS is a reliable and powerful tool for the rapid identification of *Shewanella* strains at the species level. Homology analysis of 16S rRNA gene is the most commonly used molecular identification method in prokaryotic systematics, known as the “molecular clock” of evolution [50]. However, due to the low evolution rate and high sequence conservation of the 16S rRNA gene, it is difficult to distinguish closely related species in the *Shewanella* genus [51,52]. Although the gene *gyrB* has higher resolution than 16S rRNA, there are limitations to the accurate identification of *Shewanella* species at the species level due to the lack of a unified species cutoff value and the uneven quality of sequences submitted in public databases [53–55]. Fang et al. established the method of multilocus sequence analysis (MLSA) based on several housekeeping genes [56]. The type strains of fifty-nine species were used to describe the phylogenetic relationships and taxonomy of *Shewanella*, 12 distinct monophyletic clades were defined by using MLSA to clarify the evolutionary relationships of *Shewanella* spp. Among them, the *Shewnaella* pathogens isolated from humans are concentrated in two clades, Algae (*S. algae*, *S. indica*, *S. haliotis*) and Putrefaciens (*S. putrefaciens*, *S. xiamenensis*). The *Shewanella* spp. in the same clade are more closely related, showing <4 mol% GC variation and >84% concatenated similarity [56]. Here, after updating the MLSA data of *Shewanella* type strains, split network tree has been constructed by using SplitsTree 4 program (version: 4.14.4) based on concatenated sequences of 66 *Shewanella* species type strains (Figure 3). Thirteen monophyletic clades have been clarified, and the conclusion of GC variation and concatenated similarity within the same clade consistent with that of Fang et al.

By the end of 2021, over 300 genome sequences, which belong to 63 *Shewanella* species, had been collected and collated in GenBank. Genomic data revealed that the average genome size of *Shewanella* is 4.83Mbp and the average GC content is 46.84 mol%. The genomic sizes of marine strains were significantly correlated with water depth [57]. Typically, deep-sea *Shewanella* species exhibited larger genomic sizes than their shallow-sea counterparts [57]. As the number of genomic disclosures increases, researchers can achieve accurate identification of strains using conditions of average nucleotide identity (ANI) >95% and digital DNA – DNA hybridization (dDDH) >70% [58]. Thorell et al. quantitatively assessed the genomic diversity within *Shewanella* using dDDH, and reconstructed the phylogenomic associations among *Shewanella* strains. The resulting phylogenetic reconstruction revealed two major species cluster show a big difference in G+C content [59]. At the same time, it was emphasized that as new whole genome sequences from *Shewanella* type strains become available, the complexity of the taxonomic relationships within the genus *Shewanella* will be clearly realized.

### Treatment

*Shewanella*-related infections can involve multiple parts of the human body. Based on the case reports, we summarized the treatment regimen and the resistance of the strains (Table 3). Most cases recover completely with treatment. However, there was no guideline of the antimicrobial therapy for *Shewanella* spp. infections in humans. Due to the existence of the patient’s underlying disease and the complexity of the disease spectrum, it cannot be clearly summarized what type of diseases were treated with particular antibiotic. It is certain that empirical therapy was initiated with ceftazidime, meropenem, and vancomycin et al. broad-spectrum coverage antibiotics, which could effectively control the growth of most pathogens (Table 3) [69]. Piperacillin-tazobactam was used in the initial empiric therapy for sepsis [60]. Usually, targeted treatment is according to the results of in vitro antimicrobial susceptibility testing on isolates. In the majority of cases, β-lactams, aminoglycosides, and quinolones were given to patients, which mostly had effective results. Moreover, there were great differences in drug resistance among different *Shewanella* strains. The frequent emergence of multidrug resistant (MDR) strains under the selective pressure of antibiotics makes the treatment more difficult, and clinicians need to be more cautious in antibiotic selection.

**Table 3.** The treatment regimen and drug resistance of strains summerized in the case reports.

Ref.	case num.	Year	Age/Sex	Country	Infection Type	Susceptible to	Resistant to	Antibiotic treatment	Outcome	*Shewanella*spp.
[60]	1	2022	73/M	China	C, E, F	CAZ, FEP, CN, AK, CIP, CRO, ATM, MEM, TOB, LEV, SXT	PRL (I), IPM (I), TZP (I)	ESI: TZP, CFP/SAM, MEM	D	*S. algae*
[61]	1	2021	66/M	Korea	A	AK, ATM, FEP, CAZ, CT, CN	CTX, IPM, PRL	ESI: TOB, MXF, OFX; AIP: TOB, MXF, OFX	R	*S. algae*
[62]	1	2021	66/M	Moroccan	E	AK, CT, PMB, TGC	ATM, CTX, CAZ, CIP, DPM, ETP, CN, IPM, MEM, TZP, AMC, TOB, SXT	ESI: CRO, CIP; ASA: AK	R	*S. putrefaciens*
[63]	1	2021	73/M	Caucasian	C, E	CAZ, CIP, CN, TZP, TOB, IPM, AK, AMP, AMP/SAM, CRO	KZ	ESI: FEP; ASA: CRO	R	*S. algae*
[64]	1	2021	58/M	Belize	E, H	CAZ, CIP, CN, PRL, TZP, TOB	NS	AIP: CAZ, CIP; ASA: CIP	R	*S. algae*
[65]	1	2021	46/F	Indian	H	SXT	NS	ESI: DA; ASA: LEV	R	*S. algae*
[66]	1	2020	38/M	USA	G	SXT	NS	ESI: VCM, TZP; AIP: SXT, CIP	R	*S. putrefaciens*
[67]	1	2020	86/M	Australia	E, F,	CIP, CN, CRO, MEM	KZ, AML, AMC	ASA: MEM	R	*S. algae*
[68]	1	2019	52/M	China	E, H	CAZ, FEP, CN	NS	ESI: AMP/SAM; ASA: CAZ	R	*S. algae*
[69]	2	2018	62 (avg)/M	China	C, E	TZP, CAZ, FEP, AK, CN, IPM, MEM, ATM, CAZ, FEP, AK, CN, LEV	CIP, LEV	ESI: CFP/SAM, IPM	R	*S. algae*
[70]	1	2018	69/M	Australia	D	CAZ, IPM, MEM, AK, CN, CIP	SXT, CXM, AMP/SAM, AMC	ESI: OFX, AMP, SAM, OFX, CIP; ASA: CIP	R	*S. algae*
[45]	1	2018	74/M	Bahamas	E, H	AK, SXT	CN, TOB, NET, CIP	ESI: PRL, TZP	R	*S. putrefaciens*
[71]	1	2018	27/M	Romania	F	FEP, CAZ, TZP, LEV	NS	ESI: FEP, VCM; ASA: FEP	R	*S. putrefaciens*
[20]	1	2018	34/M	Turkey	F	AK, CN, CIP, FCA	NS	ESI: ETP, VCM	R	*S. algae*
[72]	1	2017	83/F	Madagascar	B, E, H	TZP, CTX, FEP, AK	AML, AMC, TIC, PRL, ATM, MEM	ESI: CTX, AML, AMC	I	*S. algae*
[73]	1	2017	67/M	France	H	IPM, DPM, CIP, P, ATM, FEP, TZP, CAZ, CT, CN, TOB, AK.	NS	ESI: AML, CA, TZP, DA, AK; ASA: CTX, LIN, MTZ	R	*S. putrefaciens*
[74]	1	2016	65/M	China	E, H	CAZ, IPM, CN, LEV	NS	ESI: AML, CA; ASA: AML, CA, LEV	NS	*S. algae*, *S. putrefaciens*
[75]	2	2016	59 (avg)/M	Malaysia	E (case 1), F	case 1: CAZ, AK, CN, PRL; case 2: IPM, MEM, CN, AK, TZP	case 1: IPM, CIP	case 1: ESI: CLX, CAZ; ASA: CAZ, CN; case 2: ESI: CLX, CAZ	R (1); D (1)	*S. algae*
[76]	1	2016	39/M	USA	G	LEV	NS	ESI: VCM; ASA: LEV	NS	*S. algae*
[77]	1	2016	53/M	Germany	H	CRO, CIP, TZP, MEM	KZ	ESI: MEM, VCM	NS	*S. putrefaciens*
[37]	1	2016	82/F	China	E	CTX, DPM, MEM, AK	KZ, CMZ, CIP, IPM	ESI: CXM; ASA: DPM	R	*S. putrefaciens*
[17]	1	2016	65/M	Caribbean	E	CRO, CIP, CN, PRL, CAZ, MEM, SXT	NS	ESI: CRO; ASA: CRO, CIP	R	*S. algae*
[78]	1	2015	Neonatal	India	E	CIP, IPM, AK, CN, TOB, NM	CAZ, CT, PB	ESI: AMP, CN; ASA: CN	NS	*S. algae*
[79]	1	2014	50/M	India	F	TZP, CFP, SAM, FEP, CN, LEV, TGC, CT	P, CAZ, TOB, SXT, IPM, MEM	ESI: PRL, TZP	R	*S. putrefaciens*
[80]	1	2014	72/M	USA	H	PRL, TZP	NS	ESI: VCM, PRLTZP; ASA: PRL, TZP	R	*S. algae*
[81]	1	2014	27/F	China	H	AMP/SAM, CRO, FEP, IPM	KZ, CT	ESI: AMP/SAM	R	*S. algae*
[82]	1	2014	36/F	Thailand	H	C, SXT, MEM, CIP	NS	ESI: AZM, CIP	R	*S. algae*
[83]	1	2013	26/M	Korea	H	AK, CIP, IPM, PRL, ATM, AMC, SXT	NS	ESI: CRO	D	*S. algae*
[41]	1	2013	77/M	USA	G	TZP	NS	ESI: TZP, VCM	I	*S. putrefaciens*
[32]	1	2013	52/F	Thailand	H	CIP, TZP, CRO, MEM	PMB	ESI: MEM, VCM; ASA: CIP	NS	*S. algae*
[84]	1	2013	52/F	Croatia	H	TZP, CAZ, CTX, IPM, CIP	NS	PRL, CIP,	R	*S. algae*
[85]	1	2013	25/M	India	A	KZ, C, CIP, GAT, MXF, OFL	NS	ESI: GAT	NS	*S. putrefaciens*
[86]	1	2013	92/M	UK	H	CIP, TMP, AML, CN	NS	ASA: CIP	R	*S. putrefaciens*
[87]	1	2012	22/F	Brunei Darussalam	E, H	AMC, CAZ, IPM, CIP, TET	AMP	ESI: CXM, MEM; ASA: CAZ, AMC, CIP	I	*S. putrefaciens*
[88]	1	2012	43/F	Turkey	C	ATM, FEP, CAZ, CIP, CN, LEV, TZP	IPM	ESI: VCM, TZP; ASA: FEP	R	*S. putrefaciens*
[89]	1	2012	Neonatal	Puerto Rico	E	AK, CIP, CN, IPM, TOB	NS	ESI: AMP, CN; AIP: CN, AK, IPM	R	*S. algae*
[36]	1	2012	78/M	USA	E	CIP, MEM, TGC	PRL, CAZ, FEP, ATM	ESI: VCM, FEP; ASA: CN, CIP, MEM	I	*S. putrefaciens*
[90]	1	2011	82/F	Spain	E	CTX, SXT	P, AMP	ESI: CRO	NS	*S. algae*
[44]	1	2010	58/M	France	G, H	AML, TIC, PRL, ATM, CTX, CAZ, FEP, IPM, CN, TOB, AK, CT, TMP, SF, CIP	KF, FOS	ESI: PRI; AIP: CRO, AK, CIP	R	*S. algae*
[91]	1	2010	39/M	USA	C	FEP, PRL, TZP, CN, CIP, LEV, MEM	AMP/SAM	ESI: VCM, FEP; AIP: NAF, FEP	I	*S. putrefaciens*
[92]	1	2010	10/M	Croatia	B	P, KZ, PRL, TZP, AMC, CAZ, FEP, ATM, IPM, MEM, AK, CN, TOB, CIP	CXM	ESI: CXM; ASA: MEM, VCM	R	*S. algae*
[22]	1	2010	67/M	Korea	E, F, H	AMC, AK, ATM, CAZ, CIP, CRO, CTX, CN, IPM, LEV, MEM, TOB, SXT, TZP	NS	ESI: CAZ, MEM, VCM; ASA: CN, CAZ	D	*S. putrefaciens*
[93]	1	2009	21/M	Côte d’Ivoire	H	CAZ, MEM, TZP, CIP, SXT, ATM	AMC, CTX, IPM	EAI: MEM, LIN	R	*S. algae*
[26]	1	2008	42/M	China	H	ATM, AMP/SAM, TZP, CAZ, CRO, CIP, LEV, IPM, CN, AK	KF	ESI: CRO, CIP; ASA: CIP	R	*S. algae*
[94]	1	1975	82/F	Belgium	H	SXT	NS	ESI: P, C; AIP: SXT	R	*S. putrefaciens*
[95]	1	1978	60/F	South Africa	H	TET, S, C, CN, SXT, AMP, CAR, E	MET	AIP: SXT	R	*S. putrefaciens*
[33]	1	1979	63/M	USA	E, H	AMP, CAR, C, K, CN, TET, SXT	PMB	ESI: CN, C	R	*S. putrefaciens*
[96]	1	1990	75/M	Stony Brook	E	AMP, PRL, CTX, CN	KZ	ESI: KZ, CN; ASA: PRL	NS	*S. putrefaciens*
[97]	1	1991	68/M	Australia	H	P, PRL, CTX, IPM, CN, SXT	P, KF	ESI: CTX, VCM; ASA: VCM, CTX, TOB, PRL	R	*S. putrefaciens*
[98]	2	1996	74.5 (avg)/M (1), F (1)	Denmark	E (2)	AMP, PRL, CXM, CTX, CN, CIP, AMP, CAZ, PRL, CXM, CTX, CN, CIP	CAZ (I)	case 1: ESI: P, CN; ASA: CXM, CN; case 2: ESI: AMP, CN	R	*S. algae*
[99]	1	1997	61/M	N/A	H	SXT, FOX, CRO, ATM, IPM, CN, TOB, AK	NS	ESI: CRO, DA	R	*S. putrefaciens*
[100]	1	1998	67/M	Australia	H	AMC, CN, CRO, CIP	AMP, KF, TMP	ESI: P, CN, CIP; AIP: RD, SXT	I	*S. putrefaciens*
[101]	1	2002	66/M	Spain	E, H	IPM	AMP	ESI: CAZ, AK; ASA: CAZ, AK, TCP	I	*S. algae*
[102]	1	2004	27/M	Turkey	H	CAZ, FEP, CFP, CIP, CN, IPM, ATM	NS	ESI: KZ; ASA: CIP	R	*S. putrefaciens*
[103]	1	2008	76/F	China	D	TGC, CPO, CAZ, AK, SXT, IPM, TZP	CIP, TZP	ESI: FMOX; ASA: IPM	R	*S. algae*
[34]	1	2007	67/M	Japan	E	CIP	NS	ESI: IPM; AIP: CIP	R	*S. putrefaciens*
[104]	1	2007	67/F	Israel	E	AMC	NS	ESI: OMP, AMC	R	*S. putrefaciens*
[105]	1	2007	N/A	Turkey	B	AK, IPM, MEM, CIP	AMP	ASA: MEM	R	*S. putrefaciens*
[106]	1	2006	14/M	China	A	CXM, PRL, AK, CN, CAZ, CRO, MEM, IPM	AMP, KZ, SXT, CIP	ESI: AML, CXM	R	*S. algae*
[107]	1	2006	65/M	Korea	E	CAZ, FEP, ATM, AK	PRL, CN, IPM	ESI: MEM; ASA: FEP	R	*S. algae*
[43]	1	2005	45/F	Turkey	G	TIC, CAZ, CIP, IPM, CT, TOB	NS	ESI: CIP, CAZ, TOB	NS	*S. algae*
[108]	1	2004	9/F	Bartin	B	AMP, AMP/SAM, TZP, CTX, CAZ, FEP, IPM, MEM, ATM, AK, CIP	NS	ESI: AMP/SAM	R	*S. putrefaciens*
[29]	1	2004	61/F	China	E	AK, CAZ, CIP, CRO, CN, IPM, PRL, SXT	NS	ESI: KZ, CN; ASA: CFM	R	*S. putrefaciens*
[24]	1	2000	69/M	Australia	F, H	CN, TOB, AMP, CIP	NS	ESI: OFX, MTZ	R	*S. putrefaciens*
[30]	1	2000	36/M	Caucasian	E, H	CRO, CN, AMC, CIP	AML, KZ	ESI: FLX, CRO	R	*S. putrefaciens*
[109]	1	1999	64/F	Japan	E	PRL, CPO, CN, MH, LEV	IPM, KZ, ZOX	ESI: KZ, CN, LEV; ASA: MH, CPO, LEV,	R	*S. algae*
[110]	1	1998	24/F	India	D	AK, CN, CTX, PRL	NS	ESI: CN, P	R	*S. putrefaciens*
[25]	1	1983	48/M	France	B	AMP, CAR, FOX, MA, CTX, C, CN, TOB, AK, TET, CT, NA	KF, KZ, CFS	ESI: AML; AIP: CTX	R	*S. putrefaciens*

## Pathogenicity

### Morphological, physiological, and biochemical characteristics

**Figure 4.**
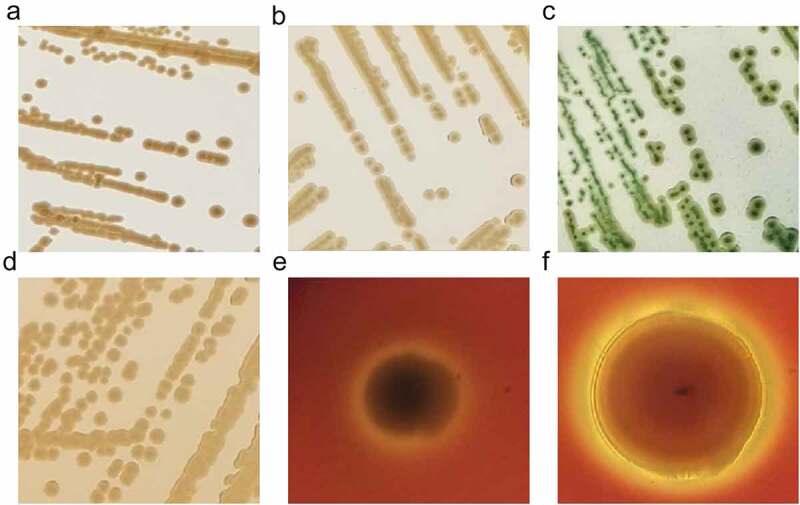
Colony morphology of *Shewanella* strains on different plates. Figure 4a, 4b, 4c and d showed the colonies on marine agar 2216, nutrient agar, TCBS and MacConkey agar, respectively, after incubating at 37 °C for 18–24 h. Figure e and f represent the growth of *S. algae* JCM 21037^T^ and *S. putrefaciens* ATCC 8071^T^ on sheep blood agar, after incubating at 37 °C for 72 h, respectively.

There is no uniform biochemical or physiological profile in the genus of *Shewanella* [2]. As marine microorganisms, *Shewanella* spp. were originally isolated with marine agar 2216E, forming round, smooth, medium-sized, and raised colonies [56]. On nutrient agar, the colonies are light brown, round and raised with intact margins. Selective media are also used for the initial isolation of *Shewanella* strains, such as thiosulphate citrate bile salts (TCBS) sucrose agar, MacConkey agar, and blood plates. Colonies on TCBS are medium-sized, smooth, raised, rounded, with colourless edges and black centres. On MacConkey agar, 1–2 mm yellow-brown colonies can be seen, while on sheep’s blood agar, some *Shewanella* species such as *S. algae* and *S. putrefaciens* can produce conspicuous β-haemolysis, and some strains can also form mucoid colonies. As researchers studied that β-haemolysis was detected in all *S. algae* strains but only in a couple of *S. putrefaciens* isolates [111,112] (Figure 4).

Derby and Hammer described the following general biochemical features of *Shewanella* spp.: Gram-negative, indole-negative, and gelatinase-, sucrose-, and maltose-positive [113]. This genus is positive for catalase and can reduce nitrate (NO_3_^−^) to nitrite (NO_2_^−^). Members of this genus are aerobic or facultative anaerobic, and some species can ferment D-glucose and *N*-acetylglucosamine to produce acid, usually without gas. Most of the *Shewanella* strains also present H_2_S-producing, urea-, arginine hydrolase-, lysine decarboxylase-negative phenotypes [12,114,115]. This article summarizes physiological and biochemical properties of seven common *Shewanella* spp. (Table 4) [12,116–120].

**Table 4.** Summary of physiological and biochemical characteristics of seven common species of the genus *Shewanella*.

Characteritics		*S. algae* [12]	*S. indica* [116]	*S. chilikensis* [117]	*S. xiamenensis* [118]	*S. putrifaciens* [12]	*S. carassii* [119]	*S. seohaensis* [120]
Type strains		JCM 21037^T^	KCTC 23171^T^	KCTC 22540^T^	JCM 16212^T^	ATCC 8071^T^	DSM 104682^T^	KCTC 23556^T^
Growth at	4°C	-	-	-	+	+	-	+
	10°C	+	+	-	+	+	+	+
	37°C	+	+	+	+	+	+	+
	45°C	-	+	-	-	-	-	-
NaCl(optimum)		0–8%(w/v) (1–3%)	0–10%(w/v) (2%)	0–8%(w/v)	0–6%(w/v) (1–2%)	0–5%(w/v) (1–3%)	0–9%(w/v) (1%)	0–6%(w/v) (2%)
pH (optimum)		5.0–11.0 (7.0–8.0)	6.5–10.0 (7.5)	7.0–10.0 (8.0)	5.0–8.0 (7.0–8.0)	7.0–8.0	5.0–8.0 (7.0–8.0)	5.0–8.0 (7.0–8.0)
Catalase		+	+	+	+	+	+	+
Oxidase		+	+	+	+	+	+	+
Nitrate reduction		+	+	-	+	+	+	+
H_2_S production		+	+	+	+	+	+	-
Urease		-	+	+	+	-	-	-
Ornithine decarboxylase		+	-	+	+	+	-	+
Gelatinase		+	+	-	+	+	+	+
Arginine dihydrolase		-	ND	-	ND	-	-	+
Lysine decarboxylase		-	ND	-	+	-	-	-
Haemolysis		+ (β)	+ (β)	+ (α)	ND	+ (β) [111,112]	+	ND
Utilization of	Glucose	+	-	+	+	-	-	+
	Sucrose	-	-	-	+	+	-	+
	Lactose	-	-	+	+	+	-	-
	Maltose	-	-	ND	+	+	-	+
	Mannose	-	-	-	ND	-	-	-
	Arabinose	-	+	ND	+	+	-	+

+: Positive; -: negative; W: weakly positive; ND, No data.

### Potential virulence factors

Pathogens must overcome several innate host defenses to attach to and colonize intestinal epithelium. Virulence factors of pathogens include the ability to invade human intestinal epithelial cells through virulence determinates, including flagella, homoserine lactones signal molecules, gelatinases, proteases and biofilm formation, which are essential for the first stage in infectious diseases [121]. Carla et al. characterized the attachment and internalization ability of *S. putrefaciens* by using human colonic carcinoma (Caco-2) cells, which shared morphological and functional features with normal small intestinal cells in post-confluent stage in vitro. The results showed that *S. putrefaciens* showed ability to attach and internalized into Caco-2 cells [121]. Members of the genus *Shewanella* have flagella, which promote motility, adhesion and biofilm formation. The mannose-sensitive haem agglutination (MSHA), and extracellular DNA (eDNA) play important roles in microbial attachment [122,123]. β-haemolysins has been found to involve in haemolytic activity, which is responsible for making *S. algae* more virulent [111]. Also, quorum sensing (QS), a cell-to-cell communication process in bacteria, has been studied in different *Shewanella* spp. [124]. In Gram-negative bacteria, the LuxI/R sensing acylhomoserine lactone (AHLs) was the most well-studied QS system [125]. *S. putrefaciens* were also able to produce AHL molecules, which could coordinate the expression of multiple virulence factors, such as biofilm development [121]. The homologous gene of LuxR has been predicted in the genomes of *S. baltica*, and the loss of this gene relates to the failure to produce AHLs. However, *S. baltica* can sense AHLs produced by other bacteria to assist QS-mediated cellular behaviours through LuxR receptor system [126]. Chitinase, lipase, protease, elastin, and alkyl sulphate enzymes produced by certain species of *Shewanella* may also associate with damage host tissues, being responsible for disruption and depletion of the mucus barrier. The genus *Shewanella* also has O-side chains or capsules with thickness varying from 20 to 130 nm, depending on different species [38]. The polysaccharide polymers on the surface of *Shewanella* are beneficial for its adhesion to solid substances and thus its infectivity [127]. In *S. oneidensis*, the biofilm facilitates microbial aggregation and then matures from a monolayer state to a three-dimensional structure, mediating by mxdABCD gene cluster and biofilm-promoting factor A (BpfA) [128,129]. Moreover, for tetrodotoxin (TTX), there are evidences that it associates with the pathogenicity of *Shewanella* [130].

Multiple virulence-specific genes, such as capsular polysaccharide biosynthesis, O‑antigen and *lasB* (vibriolysin related gene) et al. have been identified through genomes scan [131]. In the genomes of *S. algae*, some putative genes, homologous to *hlyA*, *hlyB*, *hlyD*, and *tolC* genes encoding haemolysin operon, have been identified. RTX pore-forming toxin α-haemolysin, as the product encoded by *hlyA*, could change membrane permeability and lead to cell lysis [132]. The genes related to type IV pili, which are regulated by external stimuli for directed movement, and the gene *vasF* related to VAS T6SS, provide a fresh perspective on the pathogenicity of *S. algae*. Almost all strains of *S. algae* contain *katA* gene, the product of which can decompose hydrogen peroxide, protect cells from the toxic effects of hydrogen peroxide, and enhance the bacterial colonization ability in hosts. Also, the identification of genomic islands (GIs) harbouring a suite of virulence genes and mobile elements implied that the GIs may help cross-species gene transfer and contribute to the independent acquisitions of virulence factors in *S. algae* [133]. Genes related to the spoilage metabolic pathways (including trimethylamine metabolism, sulphur metabolism, putrescine metabolism, biofilm formation and serine protease production) and to illustrate the specific QS systems were identified, providing additional evidences for its metamorphic potential and pathogenicity to aquatic animals [134]. The study by Tamez et al. proposed new candidate virulence factors including the Fur protein, OmpA, T6SS, type VI secretion effector Phospholipase A1, microbial collagenase, DNase, type IV pili, curli, twin-arginine translocation system, and ClpP [135]. Alex A et al. detected genes encoding core components of type III secretion system (T3SS) and type VI secretion system (T6SS) gene cluster, as well as the homologs of T3SS effector molecules, which aid to penetrate host mucous barriers. All structural components of T6SS were detected [136]. Virulence of pathogens depends on the activity of the T3SS injector and the effector proteins delivered to the host cell [137,138]. Previous studies revealed that VPA1328 and VopG are members of a larger family of T3SS2 effector proteins, linking to disruption of host cell survival and suppression of innate immunity in infected cells [139–141]. The VopG-like proteins have been found in *S. baltica* [142].

### Antibiotic resistance

The antimicrobial susceptibility testing panel for aerobic Gram-negative bacilli could be used to perform the antimicrobial susceptibility testing of *Shewanella* by the micro-broth dilution method. Huang et al. determined the results of susceptible (S), intermediate (I), and drug resistance (R) according to the Enterobacteriaceae standards of the American Committee for Clinical Laboratory Standards Institute (CLSI) guidelines [133,143]. Kang et al. used a disk-diffusion technique to determine the susceptibility to several antimicrobial agents [144]. The diameter of the zone of inhibition around each disk was measured and the interpretation of results was acquired according to the guidelines of the CLSI. It should be emphasized that there are no unambiguous criteria for interpretation of antibiotic resistance of *Shewanella* spp., therefore it is impossible to unequivocally compare the results obtained by different authors. Although the drug susceptibility of the genus *Shewanella* varies among different species, these bacteria were usually susceptible to the third- and fourth-generation cephalosporins, carbapenems, β-lactamase inhibitor combinations, aminoglycosides, chloramphenicol, erythromycin, aztreonam, and quinolones [3,13]. This is consistent with our findings from the summary of case reports (Table 3). *Shewanella algae* and *S. putrefaciens* are mostly susceptible to aminoglycosides, carbapenems, erythromycin, and quinolones and usually resistant to penicillin [3]. Their susceptibility to ampicillin and cephalosporins is variable [12]. It has been shown that the strains of *S. algae* strains harbouring *eptA* gene were colistin resistant [15,18,145,146], while those of *S. putrefaciens* were colistin sensitive [111]. Antibiotic resistance is on the rise in the genus of *Shewanella*. Zhao et al. investigated multidrug resistance in *S. xiamenensis* isolated from an estuarine water sample in China [147]. The results showed that the strain displayed resistance to ampicillin, aztreonam, cefepime, cefotaxime, chloramphenicol, ciprofloxacin, erythromycin, kanamycin and trimethoprim-sulfamethoxazole. As an important *Shewanella* pathogenic species, multi-drug resistant *S. xiamenensis* strains in the aquatic environment may become important reservoirs for resistance genes. Once infected in humans, it may lead to clinical treatment failure.

Bacteria of this genus are possible progenitors of many antibiotic resistance genes. The OXA-54 oxacillinase detected in *S. oneidensis* MR-1 was the progenitor of OXA-48 identified in *Klebsiella pneumoniae* [148]. *S. xiamenensis* was the progenitor of *bla*_OXA-181_ found in *K. pneumonia* [149]. *S. algae* was considered to be the origin of plasmid-mediated quinolone resistance determinant *qnrA* [150]. It was also suggested that the genus *Shewanella* is a reservoir for the MCR-4 mobile colistin resistance genes [151].

In *Shewanella* spp., the resistant genes were detected on the chromosomal regions surrounding by mobile genetic elements (MGEs) [3,45,152,153]. Numerous multidrug-resistant (MDR) and extensively drug-resistant (XDR) *Shewanella* strains have been reported in recent years [18,147,152]. Most of the *bla*_OXA-55_ genes and their variants detected in *S. algae* are located on the chromosomes, indicating intrinsic resistance to β-lactams. However, the *bla*_OXA-55_ gene identified in the MDR *S. algae* strain MARS 14 is close to a MGEs (transposon) encoding gene, suggesting *S. algae* may be a possible reservoir of this gene [18]. In the MDR *S. xiamenensis* strains, MGEs (transposon) or plasmid have been detected, containing a variety of resistance genes and conferring resistance to trimethoprim, aminoglycosides, quaternary ammonium compounds, β-lactams, chloramphenicol, sulphonamides, and tetracycline [152]. Not all *Shewanella* strains carrying β-lactam resistance genes develop a corresponding phenotype [154]. Jianhua Yin et al. discovered that ampicillin with high concentrations (>12.5 µg/ml) could induce the *blaA* gene, while ampicillin with low concentrations (<5 µg/ml) could not [155].

## Outstanding questions

First of all, the diagnostic ability of *Shewanella*-related infections needs to be strengthened. *Shewanella*-related infections were easily overlooked in clinical sets. Physicians should take care to diagnose if there exists *Shewanella* infection when patients had a history of aquatic exposure [7]. Also, clinicians need to be aware of the ability of *Shewanella* spp. to cause invasive disease, and to understand the clinical and epidemiological characteristics of *Shewanella* infections, so as to provide an earlier microbiological presumptive diagnosis and to achieve greater controllability and predictability in clinical treatment.

Secondly, methods with more rapidity and accuracy need to be established for *Shewanella* spp. identification. Considering the complex and variable biochemical characteristics of the existing *Shewanella* species as well as the discovery of new species, the traditional commercial biochemical identification systems, such as API 20E, API 20 NE, Vitek 2 GN card (bioMérieux, France), cannot correctly identify *Shewanella* strains at the species level. Therefore, it is necessary to take enough representative strains into account in experimental verification. MALDI-TOF MS, despite the most commonly used instrument in clinical bacterial detection, sometimes can cause inaccurate species-level identification due to its dependence on databases. The development of modern sequencing technology has effectively revised the phylogenetic relationships of bacterial species and improved the accuracy of species identification. However, the 16S rRNA gene or single housekeeping gene lacks discriminatory value for *Shewanella* identification at the species level. MLSA, which needs PCR of several housekeeping genes, followed by truncation and concatenation of the gene sequences, still cannot meet the requirements of clinical detection timeliness. Researchers can also achieve accurate identification of strains based on whole-genome sequence information. However, it takes a long time to obtain the genome and then to analyze it. Therefore, it is particularly important to establish rapid and accurate detection methods, such as *Shewanella* species-specific gene PCR. Besides, standard procedures for the isolation of *Shewanella* strains in food and environmental samples should also be established.

Thirdly, considering the increasing antimicrobial resistance, research on effective and judicious use of antimicrobials as well as clarification of the duration of antibiotic use in clinical treatment is needed. As an important vehicle and a reservoir of antibiotic resistance genes, the genus of *Shewanella* contains a variety of drug-resistant genetic elements, showing resistance to many drugs, including β-lactams, quinolones, aminoglycosides, macrolides and carbapenems. The use of broad-spectrum antibiotics provides continuous selection pressure to *Shewanella* strains, allowing them to evolve and become more resistant. Therefore, it is urgent to conduct drug resistance monitoring for *Shewanella* spp. and to analyze the resistance characteristics at the species level.

## Conclusion

From a clinical and aetiological perspective, this review summarized our present knowledge of the epidemiology features, clinical manifestations and pathogenic characteristics of *Shewanella* infection, highlighting that some species are associated with a wide spectrum of clinical diseases. Physicians should take care to diagnose *Shewanella* infection when patients had a history of aquatic exposure. Studies of the clinical and epidemiological characteristics of *Shewanella*-induced diseases have helped to achieve greater controllability and predictability in clinical treatment. Clinicians need to be aware of the potential of *Shewanella* spp. to cause invasive disease and should be able to provide earlier microbiological presumptive diagnosis. With the increasing incidences of *Shewanella* infection and the emerging drug resistance of *Shewanella* strains, further research is needed on how to use antibiotics effectively and judiciously, as well as on clarification of the duration of antibiotic use.
